# Microplastic uptake by birds: from observation to development of a novel seed coating to prevent bird predation of corn seeds

**DOI:** 10.1007/s11356-025-36115-x

**Published:** 2025-02-20

**Authors:** Cesare Accinelli, Veronica Bruno, Hamed K. Abbas, Chiara Morena, Vivek H. Khambhati, Wayne T. Shier

**Affiliations:** 1 Department of Agricultural and Food Sciences, Alma Mater Studiorum - University of Bologna, 40127 Bologna, Italy; 2 USDA-ARS, Biological Control of Pests Research Unit, Stoneville, MS 38776 USA; 3 Department of Medicinal Chemistry, College of Pharmacy, University of Minnesota, Minneapolis, MN 55455 USA

**Keywords:** Seed treatment, Seed predation, Wild birds, Bird repellents, Maize, Microplastic

## Abstract

The occurrence of microplastics in soil is an emerging issue that not only impacts soil ecology but also has the potential to affect terrestrial birds foraging in agricultural fields. Foraging birds may unintentionally take up soil-borne microplastics along with soil adhering to crop residues and seeds of cultivated species. Microplastic uptake by foraging terrestrial bird species, including *Pica pica* (magpie), *Corvus cornix* (hooded crow), and *Columba palumbus* (common wood pigeon), studied using camera traps, showed that the risk of MP uptake by these birds microplastics from compostable plastic bags was elevated by 27 and 78% when the microplastic particles were associated with corn seed grits or corn seeds, respectively. When these film fragments were coated with a slurry containing tannic acid, saponin, and juglone, microplastic uptake was significantly reduced. Similarly, coating corn seeds with this repellent slurry reduced seed predation by birds up to 86%, and completely wrapping corn seeds with a repellent plastic film resulted in no observed seed predation by birds. The same results were obtained when the wrapping film was replaced by a seed coating containing hairy cotton fibers which may have triggered a feather fear or repulsion response in the birds. This study demonstrated that covering corn seeds with natural repellents or hairy filaments provided complete protection from bird predation.

**Supplementary Information:**

The online version contains supplementary material available at 10.1007/s11356-025-36115-x.

## Introduction

Since the detection of microplastics (MPs) in the marine environment in the early 2000s, scientific interest on microplastic contamination has rapidly expanded (Cole et al. 2011). Although most research is still focused on the aquatic environment, recent studies have shown the abundance and significant effects of MPs in the soil ecosystem, especially in agricultural soils (Guo et al. 2020; Qin et al. 2021; Wu 2022). MPs are defined as any plastic fragments or objects with size less than 5 mm. Regardless of plastic type, MPs are divided into two major classes: (i) primary MPs, which were plastic particles that were < 5 mm when they entered the environment, such as microfibers from clothing, microbeads added to cosmetics as exfoliants, and microgranules added to detergents as foam suppressants; and (ii) secondary MPs, which are < 5-mm plastic particles generated in the environment through fragmentation of larger plastic items by natural weathering processes (Arthur et al. 2009). MPs can enter the soil mainly via field application of sewage sludge, through fragmentation of plastic mulching films, and also by the detachment of plastic-like fragments from coated seeds during planting operations (Accinelli et al. 2019; Akanyange et al. 2021; Horton et al. 2017; Rillig et al. 2017). Nowadays, plastic contamination is recognized as a global environmental issue, and serious efforts and strategies have been adopted worldwide for mitigating this environmental issue, including reducing plastic packaging, improving plastic recycling, imposing fees for single-use shopping bags, and replacing oil-based plastics with biodegradable and compostable plastics (Accinelli et al. 2022; Huerta Lwanga et al. 2017; Kasa et al. 2022).

An increasing amount of organic waste materials (e.g., food waste from households and other organic wastes) is often being collected using compostable plastic bags, and then composted in industrial composting facilities (Vinci et al. 2021). However, due to relatively short composting times (usually 6–12 weeks) and technical difficulties in separation of undesired contaminants, the industrial compost produced contains a variable amount of unseparated and partially decomposed bag fragments along with other impurities (glass, metal, and non-compostable plastic particles and fragments) (Lavagnolo et al. 2020). When the size of these film fragments from compostable plastic is less than 5 mm, they are also categorized as MPs (Accinelli et al. 2019, 2020, 2021; Akanyange et al. 2021). Due to the presence of MPs in industrial compost, application of industrial compost to agricultural fields is viewed as an important, but addressable source of MP contamination (Accinelli et al. 2020, 2022; Akanyange et al. 2021; Horton et al. 2017; Qin et al. 2021). Recent studies have shown that MPs can affect the soil ecosystem in a number of different ways, including effects on soil microbial diversity and activity (Accinelli et al. 2021; Yang et al. 2022). Soil fauna and plant growth can also be affected by MPs (Rillig and Lehmann 2020). In addition, while MP uptake by earthworms and other soil invertebrates has been observed, less information on MP exposure and uptake by birds inhabiting rural areas is available in the literature (Hodson et al. 2017; Huerta Lwanga et al. 2017). MPs routinely become incorporated into soil particles by a number of mechanisms, including soil tillage practices, the complex and dynamic crop root system, earthworms and other soil invertebrates, soil fungal hyphae, and other processes. MPs have been found tightly adherent to uncollected crop seeds and seeds of volunteer plants (Rillig et al. 2017). Birds inadvertently ingest soil particles, lead shots, and pesticide granules while feeding (Martín-Ramos et al. 2018; McArthur et al. 2012; Roy and Coy 2020). Although no specific studies focusing on this issue have been published, MPs from various sources are additional candidates to be included in the list of undesirable objects that are picked up by birds.

Seeds of many agricultural crops are typically coated with a thin plastic-like film that incorporates pesticide mixtures for protecting seeds and seedlings against plant pathogens and insects, and frequently also contains bird repellents. For some crop species, such as corn and sunflower, the absence of bird repellents in the coat mixture can result in severe yield losses (Curtis et al. 2019; Sausse et al. 2021). Anthraquinone-based bird repellents (anthracene-9,10-dione) and formulations containing a synthetic pesticide such as Methiocarb (3,5-dimethyl-4-(methylsulfanyl)phenyl N-methylcarbamate) were once widely used, but due to their unfavorable environmental and toxicological profile, they are now banned in many countries. Thus, alternative repellents are urgently needed to protect seeds from bird predation, and to protect the birds from ingesting MPs.

The present study was undertaken with the following main objectives: (1) to estimate the risk of uptake of compostable MP films by birds foraging in rural areas; (2) to investigate on the extent this process; and (3) to use this information to determine a technical solution to protect corn seeds from bird predation.

## Materials and methods

### Microplastic films and corn seeds

MP films used in the present experiments were obtained from compostable plastic bags of the commercial brand Mater-Bi® (Novamont s.p.a., Novara, Italy). These ultrathin bags (thickness 12 µm) are mainly composed of the synthetic polyester polybutylene adipate-co-terephthalate (PBAT). Squares (4 mm^2^) of ultrathin bag material were exposed to UV rays for 20 min under a laminar flow hood sterilizer, then stored in autoclaved glass tubes. To simulate food odor exposure of MP, as occurring during composting processing, a portion of these squares was incubated in flasks containing nutrient broth (VWR International, Milano, Italy). After 2 days of incubation at 20 °C with shaking, samples were dried at 40 °C. Another group of these squares was mixed into the mass of a home compost watered to its water holding capacity (Accinelli et al. 2022) and incubated at 20 °C. After 2 days of incubation, the compost was dried at 40 °C and the squares were recovered using the electrostatic approach described previously (Accinelli et al. 2020). Recovered squares were analyzed by attenuated total reflection Fourier-transform infrared equipped with a diamond sensor, ATR-FTIR (Cary 630, Agilent Technology, Santa Clara, CA, USA), and those matching the composition of the PBAT-based compostable plastic were used for the experiment.

For the bird MP uptake study, ten squares were mixed with 10 g of corn kernel grits (coarsely ground corn kernels with a size of 2–4 mm). For the seed predation study, squares were attached weakly to corn seeds (two MP squares for each seed) using small volumes (< 1–2 µL) of a 0.5% aqueous solution of methyl cellulose. The same approach was used to wrap seeds with a film of the compostable plastic. Experiments were carried out using a seed lot composed of equal amounts of seed of the following corn hybrids: PR31024 and P0900 (Corteva Agriscience Italia s.r.l., Cremona, Italy).

### Microplastic and seed coat slurry

A number of different substances and mixtures were evaluated as bird repellents (e.g., capsaicin, mint extract). After preliminary evaluations, the study focused on an aqueous slurry mixture containing the following: tannic acid (75.0%, w/w), gum arabic (1.0%, w/w), glycerol (3.0%, v/w), saponin (3.0%, w/w) and juglone (3.0%, w/w).

All chemicals were purchased from Merck Co. (St. Louis, MO, USA) except for juglone which was purchased from Tokyo Chemical Industry Co. (Tokyo, Japan). The slurry was homogenized by stirring at room temperature for 6 h before use. MP film samples were coated with the slurry manually using a 2-mm paint brush, and seeds were coated with the slurry using a small-scale seed treater machine (Hege 11, Wintersteiger AG, Ried i.I., Austria). The slurry was applied at 15 mL kg^−1^ of seeds. After slurry application, but before the slurry completely dried, rotating seeds and MP film samples were covered with short (< 1-mm length) filaments obtained from poultry feathers or cotton fibers. Feather fibers were attached manually using 0.5% methyl cellulose solution as an adhesive agent. Cotton fibers were directly added at the end of the slurry application process. Seeds were then passed through three series of 10-cm diameter brushing cylinders manufactured with neoprene. For a better adhesion between cotton fibers and seed surface, each pair of cylinders was spaced 3 mm apart and rotated at 150 rpm in opposite direction (Accinelli et al. 2023).

Seed germinability was evaluated using single germination tubes, specifically designed for testing treated seeds (Accinelli et al. 2023). Briefly, seeds were placed in the center of a 2-cm disc provided with two lateral cellulose acetate filters. The disc was then secured at the connection plane between two cylinders, which were then connected using a screw (Fig. S1). Filters were moistened, and tubes incubated in a germination chamber at 25 °C (80% relative humidity) with 12 h of light per day. Germination percentage was recorded daily, and mean germination time (MGT) calculated as follows:

$MGT=\frac{\sum \left({n}_{i}\cdot {d}_{i}\right)}{\sum {n}_{i}}$ where *n*_*i*_ is the number of seedlings present on day *i*, and *d*_*i*_ is the number of days since the beginning of the test (Ellis and Roberts 1980).

### Field evaluations

**Fig. 1:**
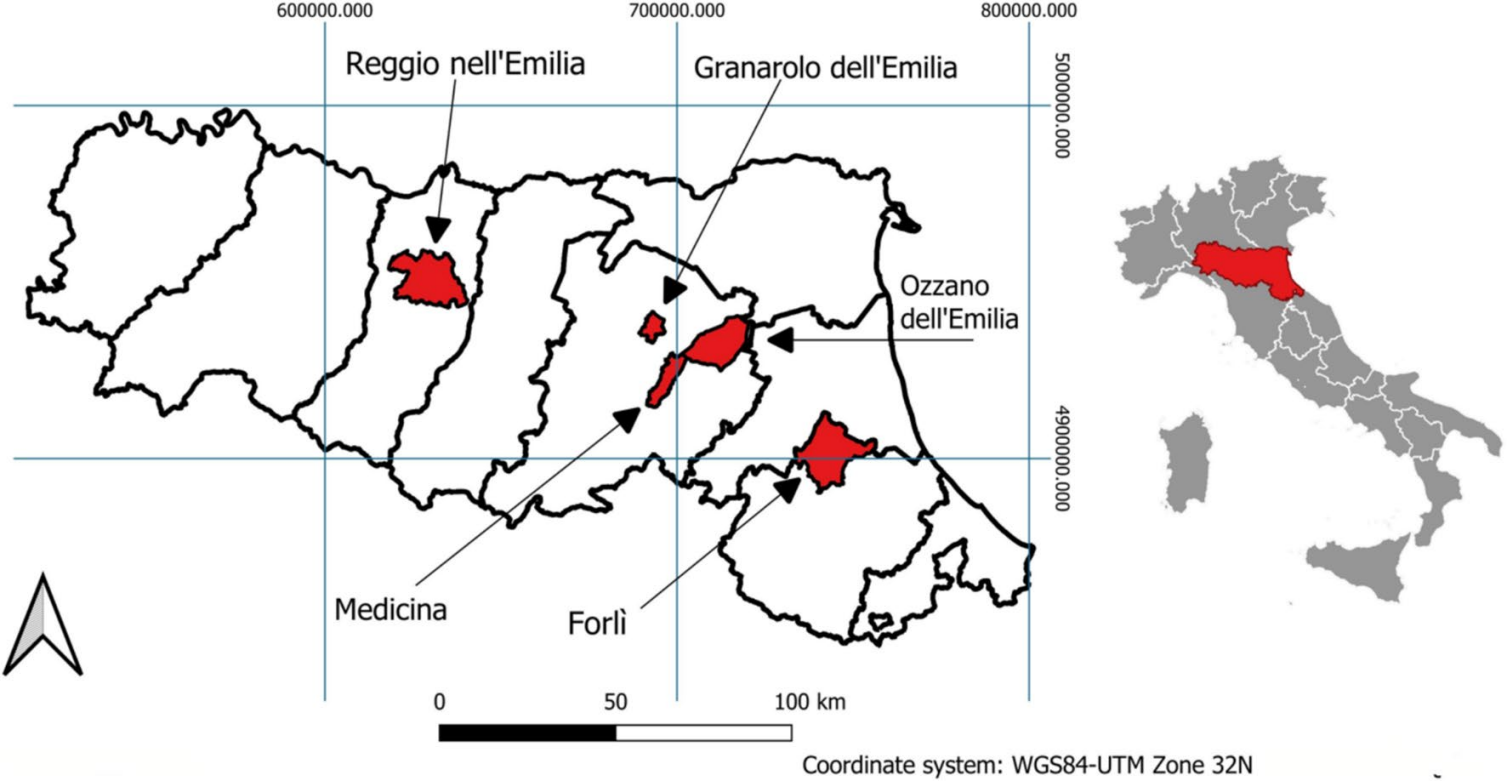
Map of the selected bird observation sites. MP uptake by birds was evaluated in five observation sites located in Emilia-Romagna, Italy

Uptake of MPs and predation of coated seeds by birds were evaluated in five sites located in rural areas of the Southern Po Valley, during 2021 and 2022. A map of observing sites is reported in Fig. 1.

At each site, testing materials were randomly placed on the ground surface of a 1-m^2^ square area (Fig. S2). Soil of the designed area was slightly compressed and/or volunteer vegetation was cut down. Each experimental site was provided with two H70 camera traps (Apeman International Co., Shenzhen, China) mounted on a metal bar 1 m above ground at a distance of 3 m from the observed area (Fig. S2). At each site, camera traps were programmed to record 1-min videos with 10-s intervals (camera 1), and 30-s videos with 10-s intervals (camera 2), for each motion-activated trigger event. Sites were inspected daily, and uptake of MPs and seeds counted for 10 days. For each observing site, an equivalent mass or number of untreated grits or seeds (control) was added to the test materials. The experiment was replicated four times for each season.

### Data analysis

Data observed for bird pick up amounts of MP, bird seed predation, and those obtained from the germination experiments are expressed as mean ± SD and analyzed by one-way analysis of variance. Comparisons between treatments were evaluated by the Tukey HSD test significance at 0.05 level using the software SPSS ver. 27.0.1.0 (SPSS Ltd., Chicago, IL, USA).

## Results and discussion

### Uptake of microplastic film particles by wild birds in the field

During the 2-year study discussed here, MP uptake by birds was evaluated in five observation sites located in different rural areas of the Southern Po Valley, Italy (Fig. 1). At these bird observation sites, the most frequent species of birds foraging on agricultural fields were magpie (*Pica pica*; MGP), hooded crow (*Corvus cornix*; HC), and common wood pigeon (*Columba palumbus*; CWP). Individuals of these three species were frequently observed pecking on bare soil searching for food and grits, particularly during the early morning. Damages to drip irrigation plastic pipes and mulching films caused by MGP and HC were documented by local farmers and directly observed during the present 2-year study. Man-made materials, including plastic and bioplastic film fragments, and nylon ropes, were found in HC nests (Fig. S3).

As shown in Table 1, the uptake of MPs in birds varied significantly (*p* < 0.05) among bird species and MP treatments. CWPs ingested 61% of MP samples that had been immersed in nutrient broth, then mixed with corn grits. For MGPs and HCs, the risk of uptake was 43 and 48%, respectively, which was significantly lower (*p* < 0.05) than with CWPs. Similar results were observed with MPs recovered from maturing compost. When non-food-like smelling MPs (control) were presented to birds, values were of 24, 26, and 31% for the two *Corvidae* species (MGP and HC) and CWPs, respectively, which was significantly lower (*p* < 0.05) consumption with respect to food-like smelling MPs. In an additional experiment, when untreated MPs were loosely attached to the surface of corn seeds, bird consumption of these samples was 77, 75, and 81%, in MGP, HC, and CWP, respectively, which was significantly higher (*p* < 0.05) consumption than when MPs were mixed with corn grits (Table 2). When MPs were associated with other nutrients (i.e., incubated in nutrient broth or compost), MGP, HC, and CWP ingested 95, 96, and 100% of seed-associated MPs, respectively. No significant differences (*p* > 0.05) were observed for MPs incubated in the two different media. These findings are consistent with increased consumption of MPs when they are associated with food, presumably as a result of odors or other sensory signals.

**Table 1:** Uptake of microplastics (MPs) mixed with corn kernel grits by three bird species, magpie (MGP), hooded crow (HC), and common wood pigeon (CWP)

Treatments	% uptake of MPs with corn grits ± SD		
	MGP	HC	CWP
Control	24.4 ± 0.7 a	26.1 ± 0.6 a	31.3 ± 0.9 b
Nutrient Broth	43.2 ± 2.4 c	48.3 ± 2.0 c	61.1 ± 2.7 d
Compost	43.0 ± 2.1 c	48.1 ± 2.3 c	56.4 ± 3.0 d
Coating	6.1 ± 2.8 e	5.1 ± 0.3 e	11.2 ± 0.9 f
Feather fibers	0.0 ± 0.0 g	0.0 ± 0.0 g	0.0 ± 0.0 g
Cotton fibers	0.0 ± 0.0 g	0.0 ± 0.0 g	0.0 ± 0.0 g

MPs were mixed with corn grits and exposed to birds at different observation sites. Before exposing to birds, MPs were subjected to the following treatments: incubated in nutrient broth or compost, coated with a bird repellent slurry, feathers or cotton fibers, or untreated controls

Values are mean ± SD of data from five observation sites. For each column, values followed by same letter are not significantly different (*p* > 0.05)

**Table 2:** Uptake of microplastics (MPs) by three bird species, magpie (MGP), hooded crow (HC), and common wood pigeon (CWP)

Treatments	% uptake of MPs with corn seed ± SD		
	MGP	HC	CWP
Control	77.1 ± 3.1 a	75.0 ± 2.8 a	81.1 ± 3.2 a
Nutrient Broth	92.3 ± 3.4 b	96.6 ± 4.1 b	100.0 ± 0.0 b
Compost	97.1 ± 3.0 b	94.5 ± 4.3 b	100.0 ± 0.0 b
Coating	57.2 ± 2.1 c	54.3 ± 1.9 c	33.8 ± 1.1 d
Feather fibers	0.0 ± 0.0 e	0.0 ± 0.0 e	0.0 ± 0.0 e
Cotton fibers	0.0 ± 0.0 e	0.0 ± 0.0 e	0.0 ± 0.0 e

MPs were loosely attached to the two sides of corn seeds. Before exposing to birds at observation sites, MPs were subjected to the following treatments: incubation in nutrient broth or compost, coating with a bird repellent slurry, coating with feather or cotton fibers, or untreated controls

MPs were attached weakly to corn seeds. Values are mean of data ± SD from five observing sites. For each column, values followed by same letter are not significantly different (*p* > 0.05)

Similar results have been reported for studies of marine birds and terrestrial raptors ingesting small-sized plastics dating back to the 1960s and this phenomenon is now well documented (Chen et al. 2021). No studies are available in the literature concerning terrestrial birds other than raptors, especially those foraging in open agricultural lands (Carlin et al. 2020; De Souza et al. 2022). Until recently, studies focusing on bird uptake of poisonous and/or synthetic materials (e.g., granular pesticides, wildlife baits) have been conducted on birds in captivity (Canavelli et al. 2014; De Montaigu and Goulson 2022; Kwon et al. 2004; Sausse et al. 2021). Difficulties in extrapolating these results to real agroecosystems have prompted the use of non-invasive approaches, including the use of camera traps, and other image and video capture devices (Contreras et al. 2022; De Montaigu and Goulson 2022; Destrez et al. 2022). Camera traps were selected for this study for this reason.

Although more specific studies are needed, focusing on ornithological and ecological aspects, data from the present experiment showed that when mixed with corn grit, more MP was taken up by CWP with respects to the two corvids. This is likely due to different diets of these bird species. Small-sized grains, such as corn grits, are preferred food for CWP. In general, MGP and HC diet is more variable and also includes insects, and other birds (i.e., young gamebirds) (Luna et al. 2024; Hadjisterkotis 2003).

As described above, compostable plastic bags intended for collecting household food wastes should be disposed of in industrial composting plants where they are held for variable periods in collection bins (e.g., 2–7 days) before being processed. Therefore, contact with food or other organic wastes lasts for at least 2–3 weeks, during which time it is expected that these packaging materials will absorb food and food decomposition volatiles into their mass, considering that compostable bags are not completely degraded during this limited period. The small-sized fragments that remain are categorized as MPs, and they can reach the soil in compost applied to agricultural fields.

Data from the present study demonstrate that when MPs are presented to birds along with food sources (e.g., corn kernel grits), the MPs are taken up by birds along with the food. These findings were extended by coating MPs with a slurry containing tannic acid and saponin, two natural compounds listed as bird repellents (Bullard et al. 1983; Bullard 1988; Filho et al. 2017; Lin et al. 2021). Juglone was also included to increase astringency (Pereira et al. 2007). When a single coated MP square was attached to each of the two sides of corn seeds (embryo and back sides), uptake of these seeds was significantly reduced (*p* < 0.05) compared to seeds which uncoated MPs were attached in the same manner. Consumption by MGP, HC, and CWP was reduced to 34, 38, and 54% of uncoated control, respectively (Table 2). Given that the size and geometric form of these seeds was not modified compared to uncoated controls, addition of these substances to the seed attaching bird repellent-coated MP squares was likely to have reduced their palatability. In another experiment, seeds were completely wrapped with compostable plastic film. Corn samples that were wrapped with untreated films were occasionally uptaken (< 6%) by MGP and HC. It was observed that in most cases, the plastic wrapping was subsequently regurgitated near the observation sites. In other cases, HCs were even able to remove wrapping film before uptake the seeds. CWP ingested 12% of wrapped seeds, and no regurgitation was observed. When the experiment was repeated with repellent-treated wrapping film, none of the wrapped seeds were taken up by the three bird species (Table 3).

**Table 3:** Uptake of coated corn seeds by three bird species, magpie (MGP), hooded crow (HC), and common wood pigeon (CWP)

Treatments	% corn seed uptake ± SD		
	MGP	HC	CWP
Control	100.0 ± 0.0 a	100.0 ± 0.0 a	100.0 ± 0.0 a
Repellent slurry coating	11.7 ± 0.8 b	13.4 ± 0.5 b	18.3 ± 0.6 c
Plastic wrapping coating	3.2 ± 0.1 d	5.6 ± 0.3 d	11.5 ± 0.4 b
Repellent plastic coating	0.0 ± 0.0 d	0.0 ± 0.0 d	0.0 ± 0.0 d
Feather fiber coating	0.0 ± 0.0 d	0.0 ± 0.0 d	0.0 ± 0.0 d
Cotton fiber coating	0.0 ± 0.0 d	0.0 ± 0.0 d	0.0 ± 0.0 d

Seeds were coated with a repellent slurry, a wrapping plastic film or with feather or cotton fibers (control: uncoated corn seeds)

Values are mean of data ± SD from five observing sites. For each column, values followed by same letter are not significantly different (*p* > 0.05)

Finding from this study indicated that MPs from compostable shopping bags and presumably from other compostable items can represent a treat to terrestrial bird foraging in agricultural fields receiving compost applications.

### Strategies for protecting corn seeds from bird predation

The strategy of completely covering seeds with a uniform coating containing repellents that was effective at reducing MP uptake was also evaluated as a strategy for preventing corn seed predation by birds foraging on agricultural lands. Corn seed coating by applying a bird repellent dispersion containing tannic acid, saponin, and juglone to the seed surface significantly reduced (*p* < 0.05) seed predation by MGP, HC, and CWP (Table 3), for which seed predation was reduced by 88, 87, and 82%, respectively. Tannic acid is classified as a primary bird repellent which works by acting as a sensory irritant and by reducing seed palatability. The effects of saponin on birds are reported in the literature, but the mechanism is still not well elucidated (Filho et al. 2017). In the present study, saponin and juglone were added to tannic acid to amplify the bitter taste effect of the coating slurry. Preliminary results showed that the two latter compounds increased the effectiveness of tannic acid–containing coatings (data not shown).

Feathers were evaluated for usefulness in achieving improved protection of seeds from predation based on basic ornithology concepts related to the roles of feathers in the interactions among birds, their predators, and rivals (Weldon 2000), including some specific cases in which feathers are directly implicated in defending birds from predators. For example, some bird species are capable of concentrating potent toxins in their plumages (Dumbacher et al. 1992). In other cases, birds have been shown to use feathers from rival species to decorate their nest to trigger a fear response in their rivals (Slagsvold and Wiebe 2021). This concept, known as the fear of feather hypothesis, was evaluated in the present study by coating corn seeds with short-length feather fibers before presentation to birds. Corn seeds coated with feather fibers were left untouched by all three bird species during the entire 1-week observation period, including by CWP, even considering that birds returned with high frequency (four to five times per day) to areas with the exposed seeds.

Corn seeds were coated with cotton filaments in a similar manner by first coating with a liquid slurry containing cotton filaments using conventional procedures in rotating drum machines, followed by drying and passing through three series of brushing cylinders attached with nylon brushes on their surfaces. Each series was composed of couples of parallel cylinders rotating in opposite directions. Application of this procedure resulted in the formation of hairy-coated seeds, which were also not eaten during the entire duration of exposure to birds (Table 3). The possibility to replace feather filaments with cotton fibers is more practicable as a commercial seed treatment approach. When MPs were coated with feathers or cotton fibers and mixed with corn grits, they were not taken up by the three terrestrial bird species (Table 1). Attaching the same MP squares to corn seeds (a single square to each of the two seed sides) resulted in a significant reduction (*p* < 0.05) of bird seed predation (Table 2).

**Fig. 2:**
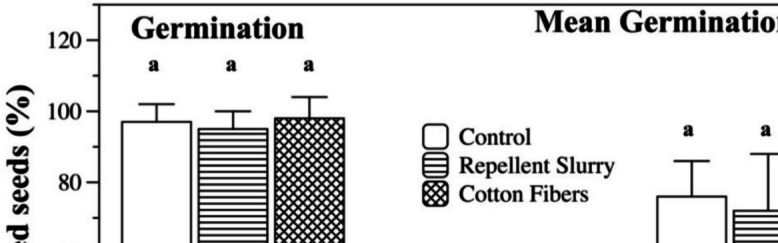
Germination (histogram on the left) and mean germination time (histogram on the right) of corn seeds that were coated with a bird repellent slurry with or without cotton fibers. Bars with same letters are not significantly different (*p* > 0.05)

Seed germination percentage and mean germination time values are summarized in Fig. 2. Coating the seeds with a bird repellent coat did not affect percent germination or mean germination time (*p* > 0.05). Application of a seed coat containing cotton fibers had no effect on germination percentage, but mean time to germination was reduced (*p* < 0.05), possibly due to cotton fibers promoting more effective absorption of water.

## Conclusions

From the introduction of industrial seed film-coating technologies in the late 1960s until the present day, the basic concept has been to cover the seeds with a thin film made of synthetic polymers formulated with chemical pest control agents. Mechanical seed planters also required that film-coated seeds should have smooth surfaces to improve their flowability. Although these procedures and approaches are well established in the seed industry, recent limitations in the use of chemical pesticides in seed treatment have created a need for new technical solutions. With a smooth surface, the absence of protective hard teguments, and finally without effective chemical repellents, the risk of coated corn seed being eaten by birds is expected to be increased and unpredictable. Also, an increasing number of seed and seed treatment companies are replacing synthetic polymers with natural, biodegradable ones, which are also less dangerous to birds, but less deterrent to predation at the same time. In the present study, the phenomenon of microplastic uptake by birds was observed, and a novel approach of protecting seeds from bird predation was developed. The concept was to apply a hairy coating to corn seeds, so they resemble seeds of some undomesticated species. These hairy-coated seeds were completely avoided by three bird species commonly encountered foraging in rural areas and agricultural fields.

In addition, the initial part of this series of studies indicated that MPs from compostable shopping bags can be uptaken by birds. Since a variable fraction of these small-size films remains after composting of organic wastes, the application of these amendments to agricultural fields poses a risk to bird foraging in rural areas.

## Supplementary Information

Below is the link to the electronic supplementary material.

Supplementary file1 (DOCX 2274 KB)

## Data Availability

Data are available from the corresponding author upon reasonable request.
