# Supplementary material for: Microplastic uptake by birds: from observation to development of a novel seed coating to prevent bird predation of corn seeds
Source: Environ Sci Pollut Res Int. 2025 Feb 20;32(10):6153–60. doi: 10.1007/s11356-025-36115-x (PMC11913899; doi:10.1007/s11356-025-36115-x)
Supplement: Supplementary file 1 — Supplementary file1 (DOCX 2274 KB) [file 11356_2025_36115_MOESM1_ESM.docx]

**Supplementary Information**


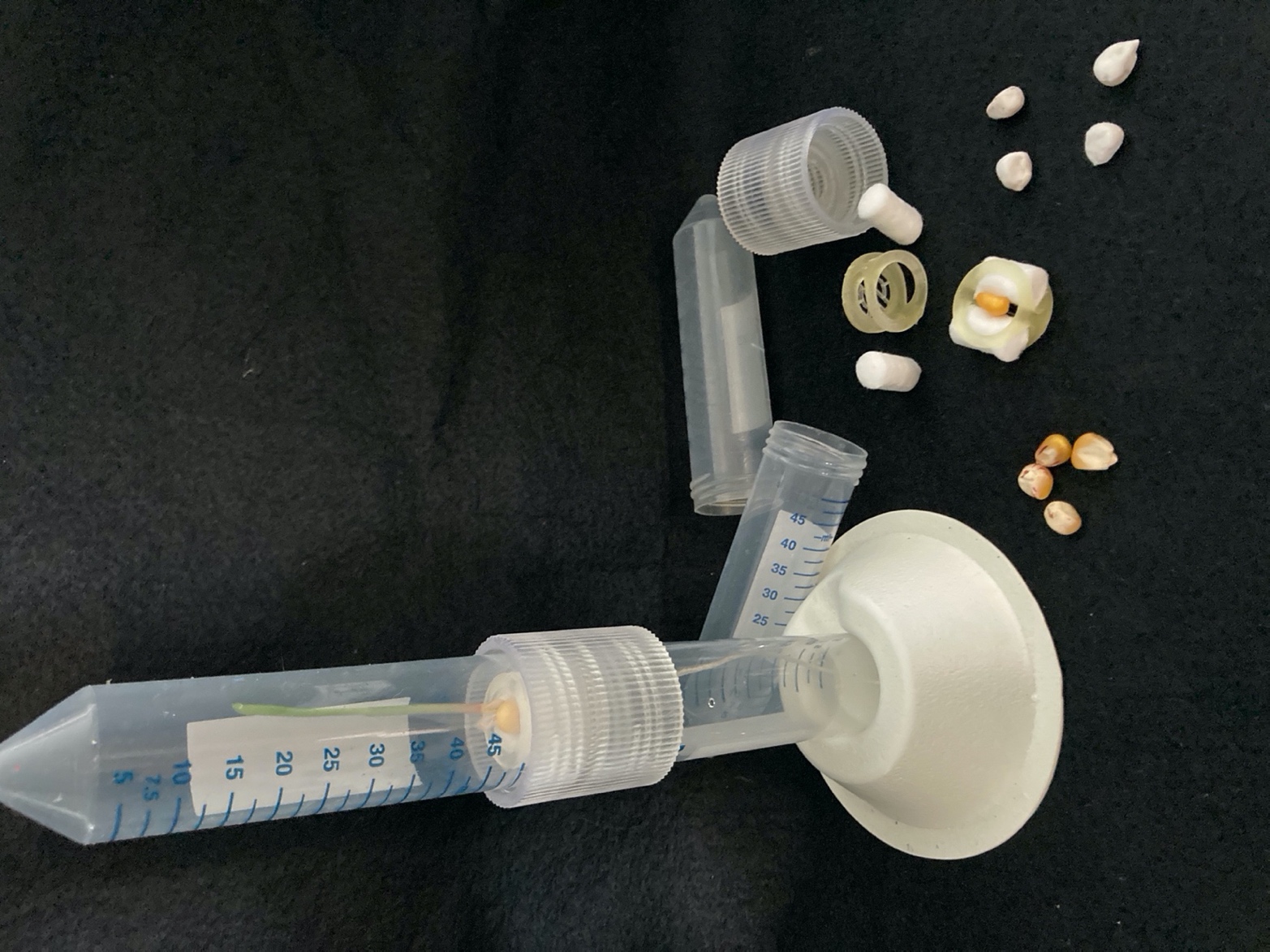


**Fig. S1** Seed germination studies were carried out using a specifically designed test tube system (left). Seeds (control seeds: left; hairy seeds: right) were secured between two cotton plugs at the junction of two test tubes held together by a threaded sleeve created with a 3-D printer.


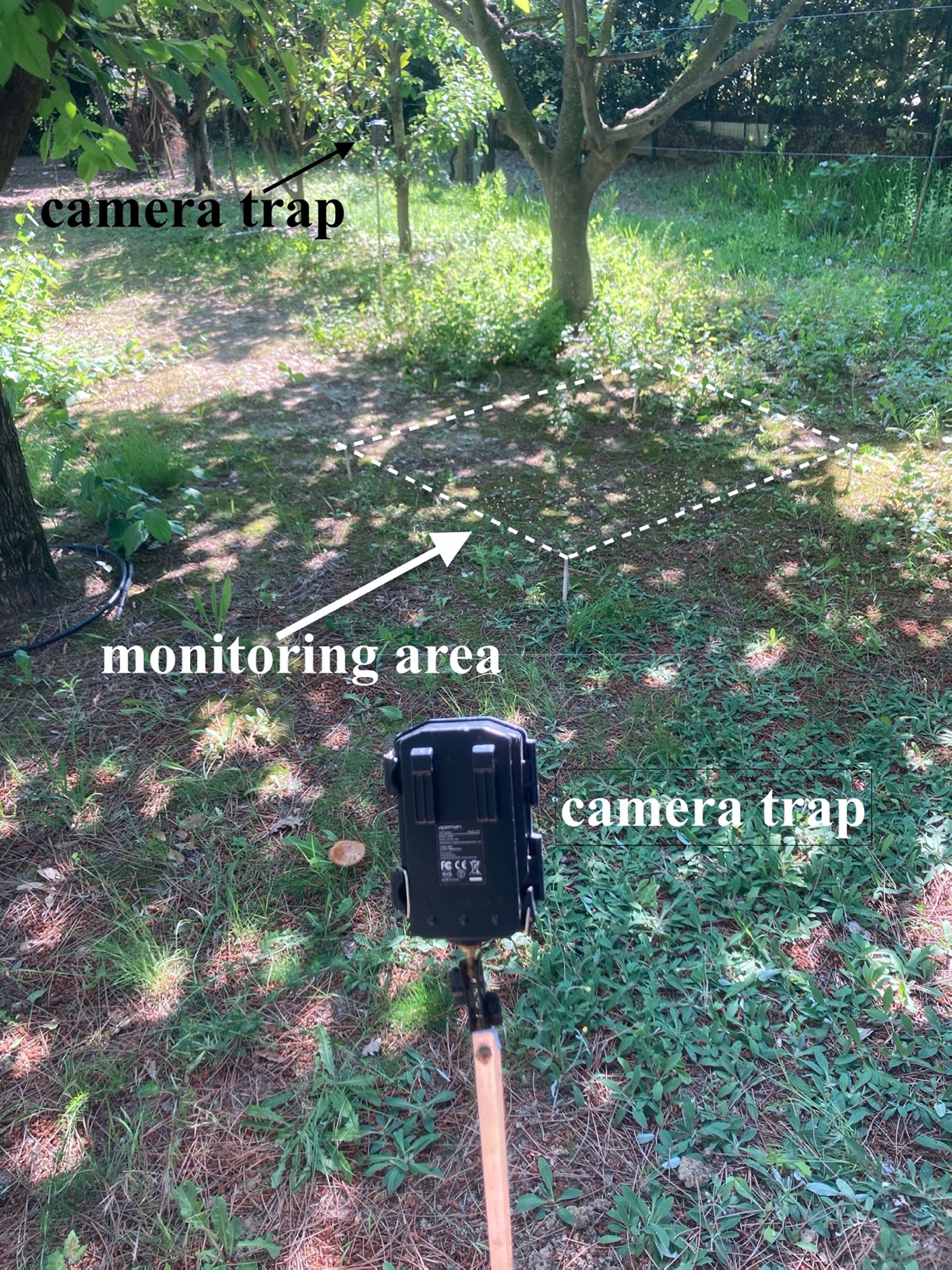


**Fig. S2** Example of a bird monitoring site. Testing materials were randomly placed on the ground of a 1-m^2^ area provided with two camera traps.


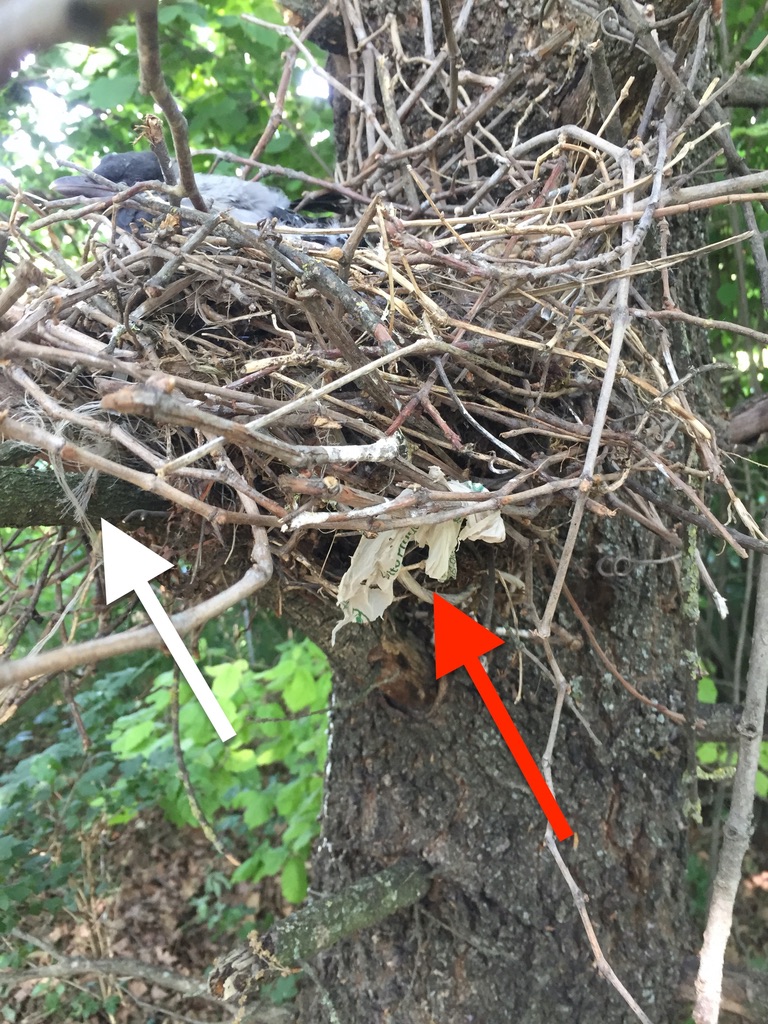


**Fig. S3** Nest of a Hooded Crow (*Corvus cornix*) with a film fragment from a compostable bag (red arrow) and nylon ropes (white arrow). The photo was taken in Cadriano, near Bologna, Italy, one of the five selected observing sites.
